# Effect of Increased Temperature on Native and Alien Nuisance Cyanobacteria from Temperate Lakes: An Experimental Approach

**DOI:** 10.3390/toxins10110445

**Published:** 2018-10-30

**Authors:** Ksenija Savadova, Hanna Mazur-Marzec, Jūratė Karosienė, Jūratė Kasperovičienė, Irma Vitonytė, Anna Toruńska-Sitarz, Judita Koreivienė

**Affiliations:** 1 Institute of Botany, Nature Research Centre, LT-08412 Vilnius, Lithuania; jurate.karosiene@gamtc.lt (J.K.) jurate.kasperoviciene@gamtc.lt (J.K.); irma.vitonyte@gamtc.lt (I.V.); 2 Division of Marine Biotechnology, Faculty of Oceanography and Geography, University of Gdańsk, Marszałka J. Piłsudskiego 46, PL-81-378 Gdynia, Poland; biohm@ug.edu.pl (H.M.-M.); anna.torunska@ug.edu.pl (A.T.-S.)

**Keywords:** global warming, shallow lakes, Europe, bloom-forming cyanobacteria, cyanotoxins, oligopeptides

## Abstract

In response to global warming, an increase in cyanobacterial blooms is expected. In this work, the response of two native species of *Planktothrix agardhii* and *Aphanizomenon gracile*, as well as the response of two species alien to Europe—*Chrysosporum bergii* and *Sphaerospermopsis aphanizomenoides*—to gradual temperature increase was tested. The northernmost point of alien species distribution in the European continent was recorded. The tested strains of native species were favoured at 20–28 °C. Alien species acted differently along temperature gradient and their growth rate was higher than native species. Temperature range of optimal growth rate for *S. aphanizomenoides* was similar to native species, while *C. bergii* was favoured at 26–30 °C but sensitive at 18–20 °C. Under all tested temperatures, non-toxic strains of the native cyanobacteria species prevailed over the toxic ones. In *P. agardhii*, the decrease in concentration of microcystins and other oligopeptides with the increasing temperature was related to higher growth rate. However, changes in saxitoxin concentration in *A. gracile* under different temperatures were not detected. Accommodating climate change perspectives, the current work showed a high necessity of further studies of temperature effect on distribution and toxicity of both native and alien cyanobacterial species.

## 1. Introduction

Anthropogenic activities are predicted to have a significant effect on climate and to promote a global temperature rise of 4 °C by 2100 [1]. Global warming more substantially will also influence Northern Hemisphere higher rather than lower latitudes [2] and will have stronger effect on inland aquatic ecosystems than on seas and oceans [3]. In Europe, the climate trends observed during the last decade show frequent events of temperature extremes and a rise throughout the continent of 1.3 ± 0.11 °C [4]. The magnitude and rate of global warming already pose a high risk of abrupt and irreversible regional changes in the structure and function of terrestrial and freshwater ecosystems [5]. According to Meehl et al. [6], longer and more intense heat waves are predicted, which will aggravate the processes even more.

Temperature is among the major determinants that effect growth rate of primary producers. Therefore, in the predicted climate scenario, the greater proportion of toxic cyanobacteria in phytoplankton assemblages is expected, as they will outcompete other taxonomic groups of algae at higher temperatures [7,8]. In temperate regions, global warming is expected to prolong and strengthen nuisance cyanobacterial blooms directly by increasing growth rates of some species or indirectly by making living environments more amenable to their proliferation (that is, longer periods with suitable temperature, higher water column stability and nutrient efflux from the sediment, lower water density, changed communities of competitors or predators, etc.) [7,9,10,11]. Cyanobacterial blooms pose a serious threat to environmental health and safety through production of hepatotoxic, neurotoxic, or cytotoxic substances and contribute to anoxia and food-web alterations [10,12,13,14]. The toxicity of a particular bloom depends on the biomass of cyanotoxin producers, the percentage of toxic genotypes in the population [15] and environmental variables that foster the toxin’s gene expression and synthesis [16,17].

In temperate regions, cyanobacterial blooms are mainly formed by species from *Microcystis*, *Planktothrix*, *Aphanizomenon* and *Anabaena*/*Dolichospermum* genera [18,19]. Non-diazotrophic *Planktothrix agardhii* preferentially occurs in deep and shallow environments and turbid waters under light-limited conditions [20] and usually forms blooms in summer and/or autumn [21,22,23], whereas the high biomass of *Aphanizomenon gracile* occurs in stratified, shallow lakes during summer. This species is also well adapted to low light conditions [21]. These cyanobacteria are well-documented producers of microcystins (MCs), saxitoxin (STX) or cylidrospermopsin (CYN) [24,25].

The other key point is that global warming not only strongly stimulates growth rates of native species but is also a driving force for adaptation and invasive cyanobacteria distribution northwards [26,27]. The dispersal corridor of invasive cyanobacterial species goes through Central Asia towards Western Europe and then throughout the Holarctic region [28]. The biggest concern is that invasions usually are reported at late stage when blooms become visible and invaders’ introduction into new habitats has already led to changes in aquatic communities and possibly in toxin composition in temperate lakes [29]. Meriluoto et al. [30] highlighted that not only native toxic cyanobacteria, but also cyanotoxin producers among alien or invasive species, should be assessed in further surveys. A particular focus should be placed on *Cylindrospermopsis raciborskii*, *Chrysosporum bergii*, *C. ovalisporum* and *Sphaerospermopsis aphanizomenoides*, which are known to be potential producers of CYN, MCs and STX [31,32,33,34].

To date, four of the seven known non-native cyanobacteria from European freshwaters [34] have been recorded in Lithuanian lakes: *C. raciborskii*, *Raphidiopsis mediterranea*, *C. bergii*, *S. aphanizomenoides* [35,36,37]. The first records of the species were determined within the last three decades [36,38,39]. *C. bergii* is known as potential CYN producer [31]. *S. aphanizomenoides* might produce CYN and possesses the *sxt* gene but the toxin production was not confirmed [32,33].

Therefore, to predict the potential of newcomers to spread out into the new regions’ bodies of water, it is important to understand drivers favouring their successful adaptation. The purpose of the present study is to assess which bloom forming native and alien cyanobacteria species are the most favoured by the increasing temperature. To address this, the effect of temperature on the growth rate of two native (*P. agardhii* and *A. gracile*) and two alien (*S. aphanizomenoides* and *C. bergii*) cyanobacteria isolated from Lithuanian freshwaters was examined. Three hypotheses were tested: i) with slight global warming, the native cyanobacteria species will have an advantage over the alien ones, whereas at a larger temperature rise the alien species establishment and blooms in the lakes of temperate zone will increase; and ii) toxic strains of cyanobacteria will prevail over non-toxic with the increase of temperature; in this way the toxicity of bloom will increase in general; iii) temperature will have impact on toxins and oligopeptides production.

## 2. Results

### 2.1. Growth Rate of Cyanobacteria Monocultures at Different Temperatures

**Figure 1:**
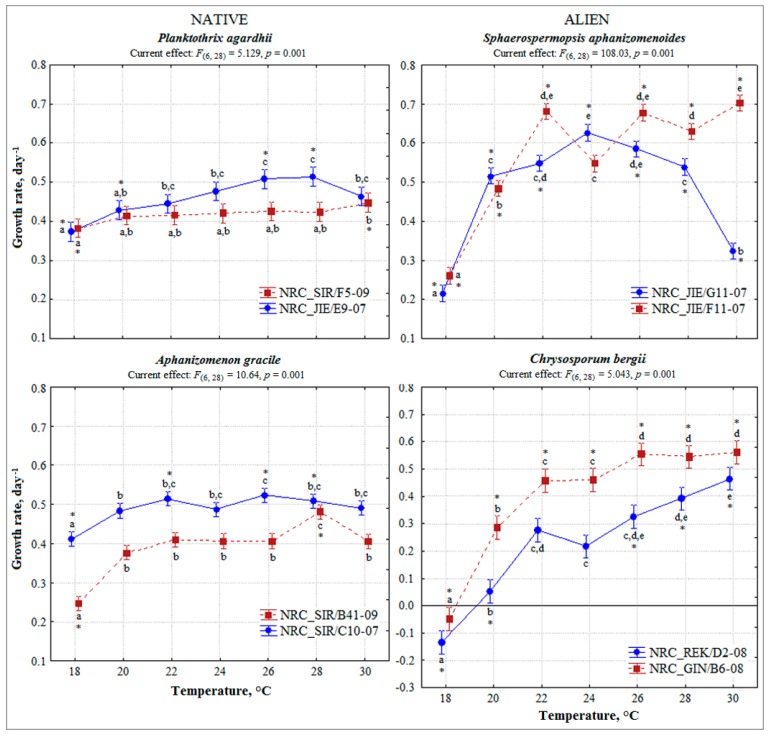
Growth rates of tested cyanobacteria strains cultured under different temperatures. Vertical bars represented 0.95 confidence intervals. Significant differences of one-way ANOVA followed by contrast test and homogeneous groups (Bonferroni test, *p* < 0.05) are indicated for each strain along temperature gradient by asterisk and similar letters, respectively. Toxic *P. agardhii* and *A. gracile* strains are in red line.

According to General linear model (GLM) analysis a significant effect of temperature (*F*_(6,161)_ = 15.83, *p* < 0.001), taxa (*F*_(3,164)_ = 15.81, *p* < 0.001), temperature and taxa interaction (*F*_(18,140)_ = 7.80, *p* < 0.001), temperature and species origin (native/alien) interaction (*F*_(6,154)_ = 5.90, *p* < 0.001) was assessed on the cyanobacteria growth rate (Table 1). In the case of native *P. agardhii* and *A. gracile*, the strains showed a wide range of temperatures favourable for the growth (18–30 °C) used in the experiments (Figure 1). Average growth rate of each strain was similar under all tested temperatures, except 18 °C and ranged from 0.41 to 0.51 and from 0.38 to 0.52 day^−1^, respectively (Figure 1). The growth rate of strains increased gradually with rising temperature and significantly differed for each strain at the temperature range 18–22 °C compared to 24–30 °C (ANOVA, contrast test *p* < 0.05). However, the growth of strains was most favoured at 20–28 °C. According to contrast test, growth rate for almost all strains of native *P. agardhii* and *A. gracile* differed significantly at 18 °C and 28 °C comparing with the rest temperatures. The strains of *P. agardhii* compared to other showed considerable potential for growth at the lowest 18 °C tested temperature.

As for alien *S. aphanizomenoides* and *C. bergii*, the strains performed differently along temperature gradient (Figure 1). Contrast test revealed significant differences in growth rate of almost all strains at tested temperatures, except *S. aphanizomenoides* strain NRC_JIE/F11-07 at 24 °C and *C. bergii* strain NRC_REK/D2-08 at 22 °C and 24 °C. *S. aphanizomenoides* strains tolerated a wide range of temperatures well 20–30 °C, similarly to native cyanobacteria. The highest growth rate was of *S. aphanizomenoides* strain NRC_JIE/F11-07 up to 0.70 ± 0.02 day^−1^ at 30 °C. Growth rate of *C. bergii* strains was most favoured at 26–30 °C with the highest value 0.56 day^−1^ (strain NRC_GIN/B6) at 30 °C and increased considerably up to 2–9 fold with warming. Generally, growth of the tested strains of alien cyanobacteria was more favoured at higher temperatures (ANOVA, contrast test for each strain at the temperature range 18–22 °C vs. 24–30 °C, *p* < 0.05).

**Figure 2:**
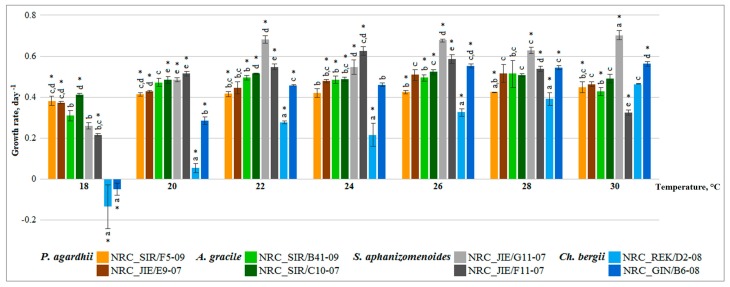
Growth rates of all native and alien cyanobacteria strains cultured under the same tested temperature. Vertical bars represented 0.95 confidence intervals. Significant differences of one-way ANOVA followed by contrast test and homogeneous groups (Bonferroni test, *p* < 0.05) are indicated for the tested strains under each temperature by asterisk and similar letters, respectively.

Additionally, variability of strains growth rate was also assessed based on comparison of the all tested strains under the same temperature. Contrast test showed that growth of all strains differed in general significantly between each other under each tested temperature (Figure 2). Also, contrast test revealed that growth rate of native cyanobacteria with the strains of alien species differed significantly (Figure 2, ANOVA, contrast test *p* < 0.001). Almost equal growth rate was found for the strains of native *P. agardhii* and *A. gracile* under each tested temperature, except at 22 °C (Figure 2, ANOVA, contrast test *p* < 0.001), while growth rate of alien *S. aphanizomenoides* and *C. bergii* strains differed significantly under each temperature, except at 30 °C (Figure 2, ANOVA, contrast test *p* < 0.001).

General linear model analysis revealed significant toxic and non-toxic strains effect regardless of temperature (*F*_(1,82)_ = 41.0, *p* < 0.001) on growth rate of *P. agardhii* and *A. gracile*. Contrast test of each strains under all temperatures showed that toxic and non-toxic strains acted differently under various temperatures (Figure 1). Growth rate of toxic strains at most of the temperatures (except *P. agardhii* at 18 °C, 30 °C and *A. gracile* at 18 °C, 28 °C) was similar (Figure 1). Whereas, growth of non-toxic strains significantly differed at wider range of temperatures *P. agardhii* (18 °C, 20 °C, 26 °C, 28 °C and *A. gracile* 18 °C, 22 °C, 26 °C, 28 °C) than toxic strains. Comparison of the tested strains under the same temperature showed that toxic and non-toxic strains significantly differed under each temperature (ANOVA, contrast test *p* < 0.004) (Figure 2). Indicated, that toxic and non-toxic strains acted differently under various temperatures and growth rate of non-toxic strains was higher than toxic strains.

### 2.2. Cyanotoxins and Oligopeptides Composition

**Figure 3:**
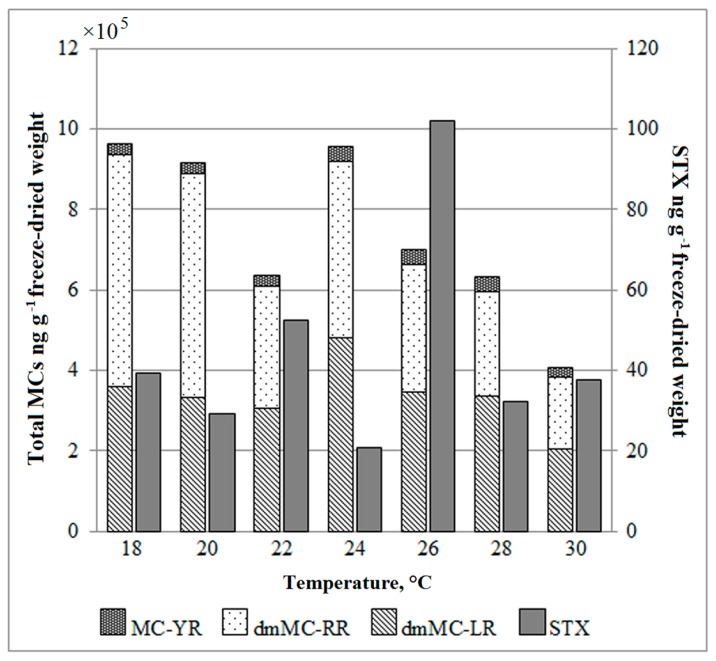
Changes in cyanotoxin concentrations in *Aphanizomenon gracile* (STX producer) and *Planktothrix agardhii* (MCs producer) grown under different temperatures. MC-YR, microcystin YR; dmMC-RR, demethylated microcystin RR; dmMC-LR, demethylated microcystin LR; STX, saxitoxin.

The toxic *A. gracile* (NRC_SIR/B41-09) strain produced STX. The concentrations of STX varied among the experimental variants from 29.4 to 102.0 ng g^−1^ freeze-dried weight (Figure 3). The highest concentration of STX was determined in the cyanobacterium grown at 26 °C and was 2–5 times higher than other culture variants. No correlation was found between temperature and growth rate with STX concentrations.

The toxic *P. agardhii* (NRC_SIR/F5-09) strain produced three variants of MCs. The dmMC-RR and dmMC-LR variants clearly prevail over MC-YR in the profile (Figure 3). The total concentration of MCs was the highest at lower temperatures (18 °C, 20 °C and 24 °C) (9.6 × 10^5^, 9.2 × 10^5^ and 9.6 × 10^5^ ng g^−1^ freeze-dried weight, relatively) and around 2.2 times lower at 30 °C (4.1 × 10^5^ ng g^−1^ freeze-dried weight). The linear regression showed a significant relationship between temperature and the total MCs concentration (*r*^2^ = 0.64, *p* < 0.05). For dmMC-RR, the correlation was even stronger (*r*^2^ = 0.81, *p* < 0.001). It is important to emphasize that temperature affected total MCs concentration but not proportion among different MC variants.

**Figure 4:**
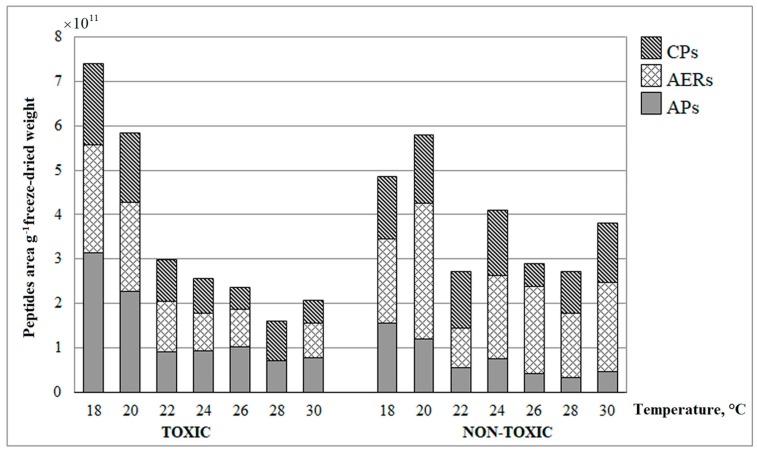
Changes in oligopeptides composition in non- and toxic *Planktothrix agardhii* strains grown at different temperatures. Oligopeptides class: CPs—cyanopeptolins, AERs—aeruginosins classes, APs—anabaenopeptins.

Among tested cyanobacteria, only *P. agardhii* strains were rich in non-ribosomal oligopeptides (NRPs) from known classes. Other strains were characterised by specific compounds; therefore, the strains of *P. agardhii* were selected for further NRPs analysis. The NRPs biosynthesis way is the same as MCs and their activity might have important environmental relevance. Three classes of oligopeptides—anabaenopeptins APs, aeruginosins AERs and cyanopeptolins CPs—were identified in both strains of *P. agardhii*. The highest relative amounts of these NRPs were recorded at the lowest temperatures (18–20 °C) (Figure 4). APs slightly prevailed in the toxic *P. agardhii* strain, whereas AERs dominated in the non-toxic strain. In the case of toxic strain, the linear regression revealed a relationship between temperature and the production of all analysed oligopeptide classes: *r*^2^ = 0.69 for APs, *r*^2^ = 0.77 for AERs and *r*^2^ = 0.74 for CPs, *p* < 0.05. For the non-toxic *P. agardhii* strain, the relationship was determined only for APs *r*^2^ = 0.75, *p* < 0.05. No relationship between temperature and the content of AERs or CPs was registered.

## 3. Discussion

### 3.1. Cyanobacteria Response to Temperature Increase

Warming may affect cyanobacteria biomass and composition of their communities in two ways. First, native cyanobacteria are expected to respond to temperature rise and some of them may outcompete the other due to species-specific environmental preferences. Second, rise in temperature will promote dispersal and establishment of non-native cyanobacteria species into temperate zone lakes, making an even more complicated interrelationship in the plankton community. These changes may also affect bloom toxicity.

Our study was focused on the response of *P. agardhii* and *A. gracile* as one of the main nuisance-dominant native species in the lakes of temperate zone [19,21,23,24,40]. *P. agardhii* and *A. gracile* can thrive in a wide range of temperatures and their populations stay dense even during cold periods (≤4 °C) [41,42]. Most frequently, these species form blooms in summer and/or autumn (September–October) at a temperature range from 10.3 to 22.6 °C in temperate lakes [21,23,37,41]. The experiments demonstrated that native *P. agardhii* and *A. gracile* were most favoured at 20–28 °C. These results are partially consistent with data published by Lürling et al. [11] and Gomes et al. [43], who found that for the European isolates of *P. agardhii*, the optimal temperature was around 27 °C and the growth rate ranged from 0.6 to 0.8 day^−1^. For *A. gracile*, the determined optimal temperature by Mehnert et al. [44], was shown to be 28 °C and 0.29 day^−1^. However, Lürling et al. [11] reported much higher values for the strains isolated from a lake in the Netherlands (32.5 °C and 0.87 day^−1^). It is important to emphasize that in the case of all tested Lithuanian isolates, optimal temperature experimentally determined in culture was similar and even higher than the temperature in which they usually develop in the lakes. This coincides with Mehnert et al. [44] observations. Likely, *P. agardhii* and *A. gracile* that are well adapted to lower temperatures in temperate lakes can have a low potential to develop at higher temperatures and to promote formation of larger biomasses in response to slight rising temperature.

**Figure 5:**
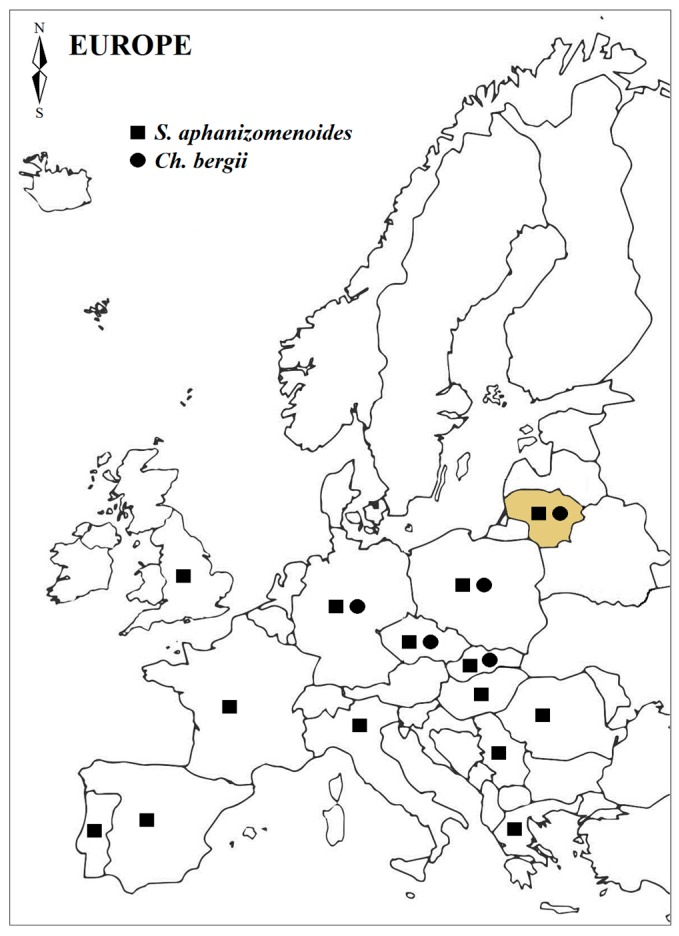
The occurrence of alien cyanobacteria, *S. aphanizomenoides* and *C. bergii*, in European freshwaters. Map was created based on information provided in Refs. [19,24,33,36,37,46,47,48,49,50,51,52,53].

Moreover, warming may favour filamentous alien species’ development over the native ones [27,44]. In this study, non-native *S. aphanizomenoides* and *C. bergii* were selected as recently dispersed in the Europe temperate zone [30], including Lithuanian waters [36,37]. Lithuania is the northernmost point of their occurrence in Europe and those species also tend to spread out and establish their populations in lakes here (Figure 5). Moreover, *S. aphanizomenoides* and *C. bergii* can coexist in the communities along with native cyanobacteria species, such as *P. agardhii* and *A. gracile* [36,37,45]. To assess the tendency of a particular non-native species to become dominant in new ecosystem, its response to the predicted changes in temperatures should be examined. Field studies of cyanobacterial communities in European water bodies reveal that both species are usually present in small amounts [33,36,37,46] but their populations, particularly *S. aphanizomenoides*, have already been established [40]. According to Budzyńska [40], *S. aphanizomenoides*’ competitive ability and success highly depends on water temperature as its domination appeared during hot weather periods.

Our experiments revealed that both alien cyanobacteria performed differently along tested temperature gradient. The optimum temperature for alien *S. aphanizomenoides* was similar to temperatures for native species 22–30 °C and for *C. bergii* reached 26–30 °C. According to Mehnert et al. [44], *S. aphanizomenoides* isolated from a German lake showed optimum temperature at 29 °C but its growth rate was almost twice as low (0.36 day^−1^) compared to our isolates. The growth rate of *C. bergii* from Lithuania reached the highest values at 30 °C, indicating that for both isolates the optimum could be even higher. In general, *C. bergii* had slow growth at lower temperatures. However, the growth rate of the Lithuanian strain at 26 °C (0.44 day^−1^) was almost twice as high compared to the German strain (0.25 day^−1^, Mehnert et al. [44]).

This study revealed that *S. aphanizomenoides* tolerated lower temperatures better than *C. bergii* and therefore is expected to invade faster. Furthermore, similar favourable wide range of temperatures for native cyanobacteria and alien of *S. aphanizomenoides* indicated that the cyanobacterium can establish in temperate lakes successfully and compete with other species. The results from culture experiments are in line with the field data of *C. bergii*’s in Lithuanian lakes. First recorded in Lake Gineitiškės in 2008 [36]), *C. bergii* was found in Lake Rėkyva (~250 km northwest from the first location) after 6 years [unpublished data]. Still, the biomass of the populations remains low up to 0.26 mg L^−1^ [36]. Growth inhibition of *C. bergii* at 20 °C and lower temperature probably slows down their proliferation. Recently *S. aphanizomenoides* appeared in Lithuanian lakes and has already built up its biomass <1.5 mg L^−1^ [37]. Mehnert et al. [44] also concluded that *S. aphanizomenoides* strains showed higher tendency to dominate than *C. bergii*. However, the summer temperature in temperate lakes was in general too low (up to 22–23 °C) [36,37] for the successful proliferation of these alien species, especially *C. bergii*.

### 3.2. Secondary Metabolites’ Variation in Response to Temperature

Many cyanobacterial blooms in the Europe are toxic and are expected to increase in frequency facing global warming [30]. The effect of temperature on cyanotoxin production may vary among cyanobacteria species but also among strains of the same species, especially those isolated from different geographical regions. Therefore, changes in the cyanobacterial community are of high concern, because the profile of the produced toxins will be altered together with the species replacement.

In general, for both cyanotoxins producing *P. agardhii* and *A. gracile* species, our results revealed significant growth differences between toxic and non-toxic strains, however did not show potential changes in dominance of toxic strains under increasing temperature. The strains of native cyanobacterial species selected for the experiment produce microcystins (*P. agardhii*) and saxitoxin (*A. gracile*). At higher temperatures, the microcystins (MCs) content in Lithuanian *P. agardhii* was lower by around 2.2 times (Figure 2). Lürling et al. [54] also revealed a decline of MCs concentration in *M. aeruginosa* at warmer temperatures. They emphasized that total MCs content could drop due to lower toxin synthesis, its higher degradation or higher association with proteins at increasing temperatures [54]. Similar to our results, Bui et al. [55] reported decreased MCs production in tropical *Microcystis* by 35–94 percent with the temperature rise. This indicate that cyanobacteria strains of different species due to increased species growth rate may accumulate less toxins with the warming events. However, the picture is not so clear, because Gianuzzi et al. [56] found that MCs cell quota of the *M. aeruginosa* strain was higher when cultured at 29 °C than at 26 °C. This is contrary to the results of other researchers who concluded that warmer temperatures favour the growth of toxic *Microcystis* strains over non-toxic ecotypes [15,57]. Davis et al. [15] showed that enhanced temperatures increased growth rates by 83 percent of toxic *Microcystis* and only 33 percent of non-toxic strains, suggesting more toxic blooms at elevated temperatures.

Variation of neurotoxin saxitoxin (STX) concentration in the Lithuanian *A. gracile* did not reveal a clear relationship with the temperature. According to Dias et al. [58], a rise in the temperature from 22 °C to 28 °C caused an approximately twofold increase of intracellular STX cell quota in *A. gracile* from Portuguese freshwaters. Cirés et al. [17] found that STX concentration also increased with temperature and was the highest at 30 °C. However, Casero et al. [59] noted that STX production was almost stable along the temperature range (15 °C, 20 °C and 28 °C) in two *A. gracile* strains from Spanish freshwaters. Unfortunately, due to different strains, media and cultivation conditions used by different researchers in their studies on the effect of temperature on cyanobacteria growth and STX production, the general conclusions and meaningful data interpretation are difficult [60].

During the current study, strains of *S. aphanizomenoides* and *C. bergii* isolated from Lithuanian freshwaters have been checked for toxin production; however, no toxins were found. Therefore, it was not possible to assess species toxicity changes in response to temperature. For example, invasive *C. raciborskii* spread in Africa, Asia, Australia and Europe but despite wide distribution in the latter continent no CYN-producing strains have been detected [61]. Likely, our studied alien species toxin production was not assessed and could be expected to increase in the future due to rise in temperature.

The variation of oligopeptides’ profile in response to temperature rise was tested only in non- and MC producing *P. agardhii* strains. Anabaenopeptins, cyanopeptolins and aeruginosins were detected in the strains. In aquatic ecosystems, these oligopeptide classes, similarly to toxins, may have negative impact on coexisting organisms. For example, AERs and CPs are potent inhibitors of serine proteases [62,63], while APs inhibit carboxy peptidase A [64,65]. The cell quota of bioactive secondary metabolites is expected to be lower at higher temperatures, due to increasing growth rate. Similar conclusions as for MCs concentrations.

Some discrepancy between the current study and published data might be expected due to different geographical location of strains isolation and due to different methodologies.

## 4. Conclusions

Global warming increases water temperature in aquatic ecosystems, which promotes cyanobacteria proliferation and opens new ecological habitats for the invaders. The experimental study revealed that favourable temperature for strains of native species of *P. agardhii*, *A. gracile* and alien *S. aphanizomenoides* was similar (20–30 °C). The strains of alien *C. bergii* appeared to be sensitive to low temperatures and mainly favoured at 26–30 °C. So, it seems that temperature rise will favour the establishment and proliferation of alien species in temperate lakes. Moreover, *S. aphanizomenoides* having the highest growth rate and widest temperature tolerance, may more easily be established into European freshwaters in the future than *C. bergii*. In the laboratory experiments, the optimum temperature for all tested species was similar and higher compared with that under which the species thrive in natural environment. The toxic and non-toxic strains of native *P. agardhii* and *A. gracile* performed differently along temperature gradient and non-toxic strains can predominate over toxic irrespective of temperature changes.

The amount of MCs and other oligopeptides (NRPs) was lower at increasing temperatures, this related to higher growth rate at warming events. In the case of *A. gracile*, the interrelation between STX productions and temperature was not clear and needs to be studied in more detailed way.

## 5. Materials and Methods

### 5.1. Cyanobacteria Isolates

Clonal cultures of freshwater filamentous cyanobacteria were used in the experiments: two native species, *P. agardhii*, *A. gracile* and two alien species in European freshwaters, *S. aphanizomenoides*, *C. bergii* (Table 2). For the study, two strains of each species isolated from Lithuanian lakes Širvys (54°59′8″, 25°13′1″), Jieznas (54°35′35″, 24°10′43″), Rėkyva (55°51′57″, 23°18′5″) and Gineitiškės (55°39′1″, 23°11′50″) in the summer months of 2014–2015 and 2017 were selected. Isolation was performed using the micropipette-washing method from the surface water samples. Then, samples were grown in modified MWC medium [66] at 20 °C under a 12:12 light:dark regime and light intensity of approximately 30 µmol m^−2^ s^−1^ using cool, white fluorescent illumination. Two strains of the native species were toxic: one strain from *P. agardhii* (NRC_SIR/F5-09) producing MCs and another strain from *A. gracile*—STX (NRC_SIR/B41-09) (Table 2). The strains were deposited at the Nature Research Centre (Lithuania). Taxonomic identification of the isolates to the species level was based on morphological criteria given in Komárek & Anagnostidis [67]. Cultures of strains were renewed before the experiments in order to reduce the number of bacteria below 1 percent of the total biomass.

### 5.2. Incubation and Experimental Design

Fourteen-day experiments in batch cultures were carried out to examine the temperature effect on the growth rate of cyanobacteria and toxins production in toxic strains. A series of the experiments were performed in the range of the temperatures from 18 °C to 30 °C with the intervals of 2 °C. Cultures were acclimated to each tested temperature for 2 days prior the experiments. The selected range is characteristic of the temperate zone of the Europe and also includes predicted gradual temperature increases in the continent in the coming decades. Temperatures of (18–22 °С) represent the average summer water temperature in European temperate lakes. The higher temperatures (>22 °C) were chosen to evaluate native and alien species’ response to the gradual warming. Experimental treatments were maintained in a modified MWC medium [66] under a 16:8 light:dark regime and light intensity of approximately 90 µmol m^−2^ s^−1^. Illumination intensity was chosen according to a given preference for native *P. agardhii* [68] and *A. gracile* [44] growth. Cultures were grown in triplicate in 100 mL Erlenmeyer flasks. In all experiments, the initial concentration of chlorophyll in the culture was the same and reached 10 ± 0.5 µg chl-*a* L^−1^. It corresponds to pre-bloom conditions in the lakes. The cultures were gently, manually mixed once a day.

### 5.3. Growth Evaluation

The growth rate (*µ*) was calculated for exponential phase (duration 6 days) based on chlorophyll *a* values. The pigment chl-*a* is useful index and rapid method for cyanobacteria biomass evaluation in cultures [69]. Chlorophyll *a* concentration was monitored every second day using a fluorometer *AlgaeLabAnalyser* (bbe Moldaenke GmbH, Schwentinental, Germany). The growth rate was calculated according to the following equation:

$$\mu =\ln\left(N_{t}-N_{0}\right)/\Delta t$$

where *N*_0_ and *N_t_*–chl-*a* values at the beginning and the end of the exponential growth phase and Δ*t* is the period of the exponential phase expressed in days [70].

### 5.4. Cyanotoxins and Oligopeptides Analysis

Before the experiment, all tested cyanobacteria strains were screened for cyanotoxins (microcystins (MCs), cylindrospermopsin (CYN), anatoxin-a (ATX-a) and saxitoxin (STX)) and non-ribosomal oligopeptides (NRPs). The detected secondary metabolites were analysed repeatedly at the end of the experiment only in the cultures of toxin-producing strains and *P. agardhii* strains with NRPs. The relative concentrations of the NRPs were expressed as the ratio of chromatographic peak area to cyanobacterial freeze-dried biomass.

The biomass of each cyanobacteria strain triplicates at each tested temperature were mixed in order to get sufficient biomass for toxins and NRPs analysis and concentrated at the end of the experiment by centrifugation 8000× *g* for 6–12 min and freeze-dried. For the extraction of MCs and other NRPs, 75 percent methanol was used as a solvent. For the extraction of STX, a mixture containing 4 mM ammonium formate buffer (pH 3.5) and acetonitrile (95:5, *v*/*v*), at a ratio of 2:3 was used. The samples were mixed by vortexing for 5 min and sonicated for 5 min in a bath sonicator (Sonorex, Bandelin, Berlin, Germany). Cyanotoxins and NRPs profiles were analysed using the liquid chromatography—yinwtandem mass spectrometry (LC–MS/MS) system according to Grabowska & Mazur-Marzec [71] and Grabowska et al. [22]. The LC–MS/MS analyses were performed using a hybrid triple quadrupole/linear ion trap mass spectrometer (QTRAP5500, Applied Biosystems, Sciex; Concorde, ON, Canada) equipped with a turbo ion spray ionization, operating in positive mode.

### 5.5. Statistical Analysis

General linear model (GLM) was applied to reveal significant effect of factors (temperature, taxa, species origin, toxic strain) and their interactions on growth rate of the tested strains. One-way ANOVA followed by contrast test and Bonferroni *p*-value correction was applied to analyse differences between (i) growth rate of the same strain under all temperatures and (ii) growth of all tested strains under the same temperature. The linear regression was used to reveal the relationship between temperature and the concentration of cyanotoxins and/or NRPs. Statistical data were processed using the STATISTICA 6.0 software package (Stat Soft. Inc., Tulsa, OK, USA).

## Figures and Tables

**Table 1:** Results of General linear model (GLM) on the effect of factors on the growth rates of tested strains.

Response Variable	Factor	Degrees of Freedom	Mean Square	*F* Value	*p* Value
Growth rate	Temperature	6	0.258	15.83	<0.001
	Taxa	3	0.312	15.81	<0.001
	Temperature × Taxa	18	0.047	7.79	<0.001
	Species origin ^1^	1	0.003	0.14	0.711
	Temperature × Species origin ^1^	6	0.082	5.89	<0.001
	Toxic strain ^2^	1	0.100	40.98	<0.001
	Temperature × Toxic strain ^2^	6	0.001	0.76	0.606

^1^, native/alien; ^2^, toxic and non-toxic strains of *P. agardhii* and *A. gracile*.

**Table 2:** Cyanobacteria strains used in the experiments.

Species		Strain	Lake and Year of Isolation of the Strain	Cyanotoxins	NRPs
Native	*P. agardhii*	NRC_SIR/F5-09	Širvys, 2014	MCs	+
		NRC_JIE/E9-07	Jieznas, 2015	-	+
	*A. gracile*	NRC_SIR/B41-09	Širvys, 2015	STX	-
		NRC_SIR/C10-07	Širvys, 2015	-	-
Alien in Europe	*S. aphanizomenoides*	NRC_JIE/G11-07	Jieznas, 2015	-	-
		NRC_JIE/F11-07	Jieznas, 2015	-	-
	*C. bergii*	NRC_REK/D2-08	Rėkyva, 2015	-	-
		NRC_GIN/B6-08	Gineitiškės, 2017	-	-

NRPs, non-ribosomal oligopeptides; MCs, microcystins; STX, saxitoxin; +, detected; -, not detected.
